# Comprehensive analysis of mitophagy-related genes reveals prognostic signatures in breast cancer: based on immune landscapes and treatment target predict

**DOI:** 10.3389/fimmu.2026.1740830

**Published:** 2026-02-05

**Authors:** Hejia Zhao, Wanying Zhang, Yiheng Du, Zheng Kuang, Yilan Tan, Yanjun Chen, Yujie Ma, Fei Zou

**Affiliations:** 1 Department of Occupational Health and Medicine, Guangdong Provincial Key Laboratory of Tropical Disease Research, School of Public Health, Southern Medical University, Guangzhou, China; 2 Department of Molecular Biology, Graduate School of Medicine, Nagoya University, Nagoya, Japan; 3 Department of Occupational Health and Occupational Medicine, Guangdong Provincial Key Laboratory of Tropical Disease Research, School of Public Health, Southern Medical University, Guangzhou, China

**Keywords:** breast cancer, early diagnosis, immune cell infiltration, mitophagy, prognostic biomarkers

## Abstract

**Background:**

Breast cancer (BC) is a common malignant tumor with high incidence and mortality rates. Mitophagy refers to a selective form of autophagy that is believed to be closely related to the occurrence and progression of BC. Identifying the mitophagy-related sites associated with BC can help us gain a deeper understanding of the underlying mechanisms of BC, laying the foundation for early diagnosis and effective treatment of BC.

**Method:**

RNA-seq expression data of BC were obtained from the GEO and TCGA databases. Differentially expressed genes were intersected with mitophagy-related genes from GeneCards to identify BC-associated mitophagy genes. Prognostic biomarkers were screened using Kaplan–Meier (K–M) survival and ROC analyses. Based on mitophagy-related gene expression and survival data, BC patients were classified into high- and low-risk subgroups for immune infiltration and GSEA analyses. Finally, IHC data from the HPA database and *in vitro* experiments, including siRNA-mediated knockdown, Western blot, CCK-8 proliferation assay, confocal microscopy and drug prediction were performed to validate the expression and biological functions of candidate biomarkers PBK and NEK2.

**Result:**

Through dual validation of K-M survival analysis and ROC diagnosis-treatment efficacy analysis, we ultimately identified 9 mitophagy-related prognostic biomarkers for BC, and found their expression was significantly upregulated in BC tissues. In addition, the results showed that the degree of immune infiltration in the low-risk subgroup was considered higher than that in the high-risk subgroup. Inhibition of PBK and NEK2 will have an inhibitory effect on the proliferation of BC cell. Furthermore, clinicopathological analyses confirmed a genuinely higher risk in the high-risk subgroup, with PBK and NEK2 independently associated with risk stratification.

**Conclusion:**

This study elucidated the prognostic value, immune microenvironment characteristics, and molecular mechanisms of mitophagy in BC, and identified nine mitophagy-related biomarkers. Among them, PBK and NEK2 were experimentally confirmed to promote tumor cell proliferation, providing novel insights for early diagnosis and therapeutic strategies in breast cancer.

## 1 Introduction

Breast Cancer (BC) is one of the three most common cancers in the world, along with lung cancer and colon cancer, and is the most common malignant tumor in women (1). It is estimated that in 2020, there were 2.3 million new cases of breast cancer and more than 685000 deaths (2). A variety of adverse risk factors can induce the occurrence of breast cancer. Research shows that aging, family history, reproductive factors, endogenous and exogenous estrogen content, excessive drinking and excessive fat intake are all possible risk factors for breast cancer (3–8). Statistics show that one in eight to one in ten women will suffer from breast cancer in their lifetime. In recent years, the mortality rate of breast cancer in North America and the European Union (EU) has declined, mainly due to early detection and effective systematic treatment (9). Although many studies have been conducted on BC and a small number of biomarkers have been applied in its early diagnosis (10), the underlying molecular mechanism of its pathogenesis is still unclear. Therefore, it is crucial to search for and explore new potential prognostic biomarkers in BC patients and to elucidate the underlying molecular mechanisms of BC.

In view of the extremely high malignancy, the high risk of metastasis, and the poor prognosis of late-stage BC, identifying and determining effective molecular biomarkers is of great significance for the early diagnosis and treatment of BC (11). The existing research results show that human epidermal growth factor receptor 2 (HER2), cell proliferation related nuclear protein (Ki-67), estrogen receptor family (ERs), progesterone receptor family (PRs), etc. are important proteins involved in the pathogenesis of BC (12–15). In addition, due to the improvement in molecular detection technology, a variety of MicroRNAs such as Let-7, miR-34c, miR-200c, miR-183, miR-16 and miR-203 can participate in multiple stages of breast cancer by influencing several cell and molecular targets (i.e. Sp1, Wip1, EMT and BMI 1), and are also considered to have potential value in the diagnosis and monitoring of breast cancer (15–17). Because of the limitations of various detection and diagnostic techniques for BC, such as imaging detection techniques, which may be constrained by high prices and a lack of sensitivity and specificity (18); The use of biomarker detection methods can achieve early and accurate identification of BC, which has profound significance for the prognosis on BC.

Mitophagy (also known as Mitochondrial Autophagy) is a selective form of megaautophagy and the only known pathway to remove completely, damaged, and dysfunctional mitochondria. It is an important pathway for cells to respond to stress and plays a crucial role in maintaining cellular homeostasis (19, 20). However, the role of mitophagy in cancer cells is complex and diverse (21). Research has shown that although mitophagy is associated with tumor inhibition in the early stages of tumor development, it may promote tumor growth in the later stages. More recently, mitophagy has been recognized as a critical modulator of the tumor immune microenvironment (22). Dysregulated mitophagy influences mitochondrial-derived danger signals, such as mitochondrial DNA and reactive oxygen species, thereby shaping innate immune signaling pathways and altering immune cell recruitment and activation (28, 33). Moreover, aberrant mitophagy in tumor cells has been implicated in immune evasion by affecting antigen presentation, immune cell infiltration, and the balance between immune activation and immunosuppression within the tumor microenvironment (30). Given the dual and immunoregulatory roles of mitophagy in cancer progression, elucidating the mechanisms by which mitophagy contributes to breast cancer development and immune landscape remodeling, as well as identifying mitophagy-related prognostic biomarkers, may provide important insights for early diagnosis, risk stratification, and personalized therapeutic strategies in breast cancer (36).

In this study, we obtained three BC datasets (GSE5364, GSE29044, and GSE42568) from the GEO database, identified common differentially expressed genes, and intersected them with mitophagy-related genes from the GeneCards database to obtain 70 candidate genes. A protein–protein interaction (PPI) network was then constructed to identify 12 hub genes with strong correlations. After Kaplan–Meier (K–M) survival and ROC analyses, nine mitophagy-related prognostic genes were ultimately identified and their expression levels were validated using immunohistochemistry (IHC) data. Furthermore, based on the TCGA-BRCA dataset, BC patients were classified into high- and low-risk subgroups according to mitophagy gene expression profiles, and differences in pathway enrichment and immune infiltration were analyzed using GSEA and MCPCounter algorithms. In addition, to further validate the functional significance of the key mitophagy-related biomarkers, *in vitro* experiments including siRNA-mediated knockdown, Western blot, CCK-8 proliferation assay, and confocal microscopy were performed for PBK and NEK2, confirming their oncogenic roles in promoting breast cancer cell proliferation, drug sensitivity analysis was conducted to explore the relationship between the expression of PBK, NEK2 and therapeutic compounds. Collectively, our research provides a comprehensive bioinformatics and experimental framework for elucidating the role of mitophagy in BC, laying a molecular foundation for early diagnosis, prognostic evaluation, and targeted therapy.

## 2 Materials and methods

### 2.1 Acquisition of raw data

We applied the common datasets GSE5364 (23), GSE29044 (24), and GSE42568 (25) from the Gene Expression Omnibus (GEO https://www.ncbi.nlm.nih.gov/geo/) database, and obtained gene expression data and corresponding clinical data from the TCGA-BRCA project in The Cancer Genome Atlas (TCGA https://portal.gdc.cancer.gov/) database.

### 2.2 Download of mitophagy-related sites

We searched and extracted mitophagy-related genes from the GeneCards database (https://www.genecards.org/) using the keyword “mitophagy.” The GeneCards relevance score integrates evidence from curated databases, functional annotations, and published literature to quantify gene–process associations. Genes with a relevance score > 1 were selected to retain those with moderate to strong evidence supporting their involvement in mitophagy, as commonly adopted in previous bioinformatics studies, while reducing potential noise from weakly associated genes. This threshold allowed us to maintain sufficient gene coverage for network construction and survival analyses while minimizing the inclusion of weakly supported candidates.

### 2.3 Differential expression genes analysis

For three data sets from the GEO database (GSE5364, GSE29044, GSE42568), we used GEO2R to identify differentially expressed genes and calculated the significance FDR for each gene using the P.adjust function. Finally, based on the significant differential genes (adj.P<0.05, Log Fold Change>1.5) and the availability of data, we achieved visualization of volcano and box plots.

### 2.4 Functional enrichment analysis

We selected significant differentially expressed genes from each of the three datasets in the GEO database. Use the Metascape database (https://metascape.org/) to perform correlation and enrichment analysis on three groups of significantly differentially expressed genes. We used the following criteria for the analysis: (1) Three gene lists were used to input genes in sequence; (2) For GO annotations, three main types of gene functions were extracted: biological processes (BP), molecular functions (MF), and cellular components (CC); (3) In functional enrichment analysis, the default parameters for filtering are: minimum overlap of 3, enrichment factor of 1.5, and P-value of 0.01. Meanwhile, the Metascape database also constructed and visualized protein-protein interaction networks at input sites based on STRING software.

### 2.5 Construction of protein-protein interaction networks

Firstly, we extracted significantly differentially expressed genes (DEGs) that were consistently identified across three GEO datasets (GSE5364, GSE29044, and GSE42568), resulting in a total of 177 common DEGs. These genes represent robust BC–associated transcriptional alterations shared among independent cohorts. Subsequently, the 177 DEGs were intersected with mitophagy-related genes retrieved from the GeneCards database to identify genes that are both functionally associated with mitophagy and transcriptionally dysregulated in BC. This integrative filtering strategy yielded 70 overlapping genes, which were defined as *mitophagy-related genes* in the context of BC and were subjected to subsequent analyses in this study. After that, import 70 intersection sites into the STRING database (https://string-db.org) and generate a PPI network. When constructing a PPI network, PPI network pairs with a moderate confidence score > 0.4 were exported, and then visualized using Cytoscape (version 3.8.2). Among them, we applied Molecular Complex Detection (MCODE http://apps.cytoscape.org/apps/mcode) to find densely connected areas and subnets within the main network; In addition, five topological analysis methods of cytoHubba (https://apps.cytoscape.org/apps/cytohubba) were applied to rank and screen hub sites using gene attributes.

### 2.6 Mutation correlation analysis

We use the Sangerbox database (http://www.sangerbox.com/tool) to obtain somatic mutation data related to BC samples in “maf” format from the TCGA database. We used the “Maftools” software package in R software to draw the waterfall diagram of somatic mutation and visualize the mutation sites.

### 2.7 Screening for potential prognostic related sites

In order to screen potential prognostic related sites for BC among 70 Mitophagy related sites, we extracted 12 sites from the “MCODE1” network with the highest score. Use the Kaplan Meier Plotter database (http://kmplot.com/analysis/) to draw the K-M survival curve, and the ROC Plotter database (www.rocplot.org) to draw the ROC curve; Analyze each site and further screen 12 Mitophagy related sites based on K-M survival data and ROC-AUC (26).

### 2.8 Consistency clustering analysis

In the Sangerbox database, we identified Mitophagy-related tumor subtypes (High-Risk, Low-Risk) by consistency clustering using the “ConcentrusClusterPlus” software package from R software. Then, we evaluated the clustering results with k between 2 and 10 and repeated the process of obtaining the ideal number of clusters 1000 times to ensure stable results and achieved data visualization.

### 2.9 Gene Set Enrichment Analysis method

We applied Gene Set Enrichment Analysis (GSEA) to detect whether the enrichment of GO and KEGG pathways is statistically significant in high-risk and low-risk subtypes. The transcriptome data from TCGA database were divided into two groups in high-risk and low-risk subtypes and imported into GSAE software. Both p<0.05 and FDR<0.25 are considered criteria for significant gene sets (27).

### 2.10 Tumor microenvironment and immune infiltration analysis

In order to identify the difference of immune characteristics between high risk and low risk subtypes, we loaded the transcriptome expression data into CIBERSORT database (https://cibersort.stanford.edu/), and repeated the results 1000 times to determine the relative percentage of 22 immune cell types. To further confirm the accuracy of the data, we used the MCPCounter Package from Rstudio to detect infiltration differences in 8 immune cell families and two stromal cell families in two tumor subtypes. Finally, we analyzed and calculated the differences in the scores of Stromal score, Immune score, and ESTIMATE score between the two subtypes using the ESTIMATE database (https://bioinformatics.mdanderson.org/estimate/).

### 2.11 Tissue protein expression verification

The immunohistochemical (IHC) images of normal human tissues and BC tissues were obtained from human protein profiles (HPA, https://www.proteinatlas.org/). Used to identify the protein expression differences of the ultimately screened 8 Mitophagy-related sites between human normal tissue samples and BC samples.

### 2.12 Drug sensitivity analysis of characteristic DEGs

The NCI-60 cell line panel is a drug efficacy screening developed by the Developmental Therapeutics Program (DTP) of the National Cancer Institute (NCI) in the United States. Thousands of compounds have been applied to the NCI-60. CellMiner (https://www.nci.nih.gov/cellminer/) is a web-based genomic and pharmacological toolset for exploring transcriptional and drug profiles in the NCI-60 cell line panel. Based on the corresponding data from CellMiner, the Corrplot R software package and Spearman method (p < 0.05) were used to analyze the relationship between the expression of PBK, NEK2 and drug sensitivity.

### 2.13 Cell culture

MDA-MB-231 and MCF-7 was purchased from the American Type Culture Collection (Manassas, VA, USA), and has been authenticated for mycoplasma contamination and short tandem repeat profile analysis. In an incubator set at 37 °C with 5% CO_2_, cells were grown in DMEM combined with 10% fetal bovine serum and 100 U/ml penicillin-streptomycin combination (Gibco, USA).

### 2.14 Antibodies and reagents

Antibodies against PBK(4942T), a-Tublin(3873T), b-Actin(4967T) were acquired from Cell Signaling Technology (Danvers, MA, USA); Antibodies against NEK2(ab279717) were acquired from Abcam (Cambridge, MA, USA). Ki67(9129S) was acquired from Cell Signaling Technology (Danvers, MA, USA).

### 2.15 Western blot

The cell lysis assay kit (KGP 2100) was purchased from Keygen Biotech (Nanjing, China). Cells were added to lysis buffer and lysed on ice for 5 minutes. The cells were scraped off using a cell suspension apparatus and transferred to a 1.5 ml centrifuge at 4°C for 15 minutes at 13000 rpm. The supernatant was tested for protein concentration using a detergent compatible Bradford protein assay kit (P0006 C). For protein extraction of liver cancer tissue and xenograft tumor tissue, 300 μ l of whole protein lysate was added every 10 mg of tissue, and thoroughly ground in a protein homogenizer. The supernatant was centrifuged under the same conditions as described above to detect protein concentration. The proteins were separated by sodium dodecyl sulfate polyacrylamide gel electrophoresis, and then moved to the polyvinylidene fluoride (PVDF) membrane. Prepare PVDF membrane by blocking it with 5% BSA, and then add primary antibody overnight at 4°C; Add secondary antibody at room temperature for 1 hour, scan with Li COR Odyssey infrared imaging system, and finally analyze with Image J software.

### 2.16 Cell proliferation

5x10^4^ cells were cultured in 96-well cluster plates. CCK-8 reagents were used to dilute at a ratio of 1:9; add CCK-8 reagent 100 μl per well in the dark for 2 h in a 37°C, finally read the absorbance at 450 nm wavelength.

### 2.17 Immunofluorescence analysis

Cells were seeded in confocal dishes at a density of 1 × 10^5^ cells per well and treated with the indicated drugs or reagents. After treatment, cells were fixed with 100% methanol at −20°C for 15 min and permeabilized with 0.1% Triton X-100 for 5 min. Following PBS washes, cells were blocked with 5% bovine serum albumin (BSA) in PBS at room temperature for 1 h. Subsequently, cells were incubated with primary antibodies at 4°C overnight. After thorough PBS washing, cells were incubated with fluorescently labeled secondary antibodies at room temperature for 2 h. Nuclei were counterstained with DAPI. Fluorescence images were captured using an Olympus FV1000 confocal laser scanning microscope (Tokyo, Japan). The degree of protein colocalization was quantified using Image-Pro Plus software, which calculated the overlap coefficient between the two fluorescence signals.

### 2.18 Statistical analysis

All statistical analyses were performed using SPSS software (version 21.0; IBM Corp., Armonk, NY, USA). Continuous variables were presented as mean ± standard deviation or median (interquartile range), as appropriate, and compared using the Student’s t-test or Mann–Whitney U test. Categorical variables were analyzed using the chi-square test or Fisher’s exact test. Survival differences were evaluated using Kaplan–Meier analysis and compared by the log-rank test. Univariate and multivariate Cox proportional hazards regression analyses were conducted to identify independent prognostic factors, with hazard ratios (HRs) and 95% confidence intervals (CIs) reported. Logistic regression analysis was used to assess the independent association of PBK and NEK2 expression with high-risk classification, and odds ratios (ORs) with 95% CIs were calculated. All statistical tests were two-sided, and a P value < 0.05 was considered statistically significant.

## 3 Result

### 3.1 Identification of differentially expressed genes in BC datasets

**Figure 1:**
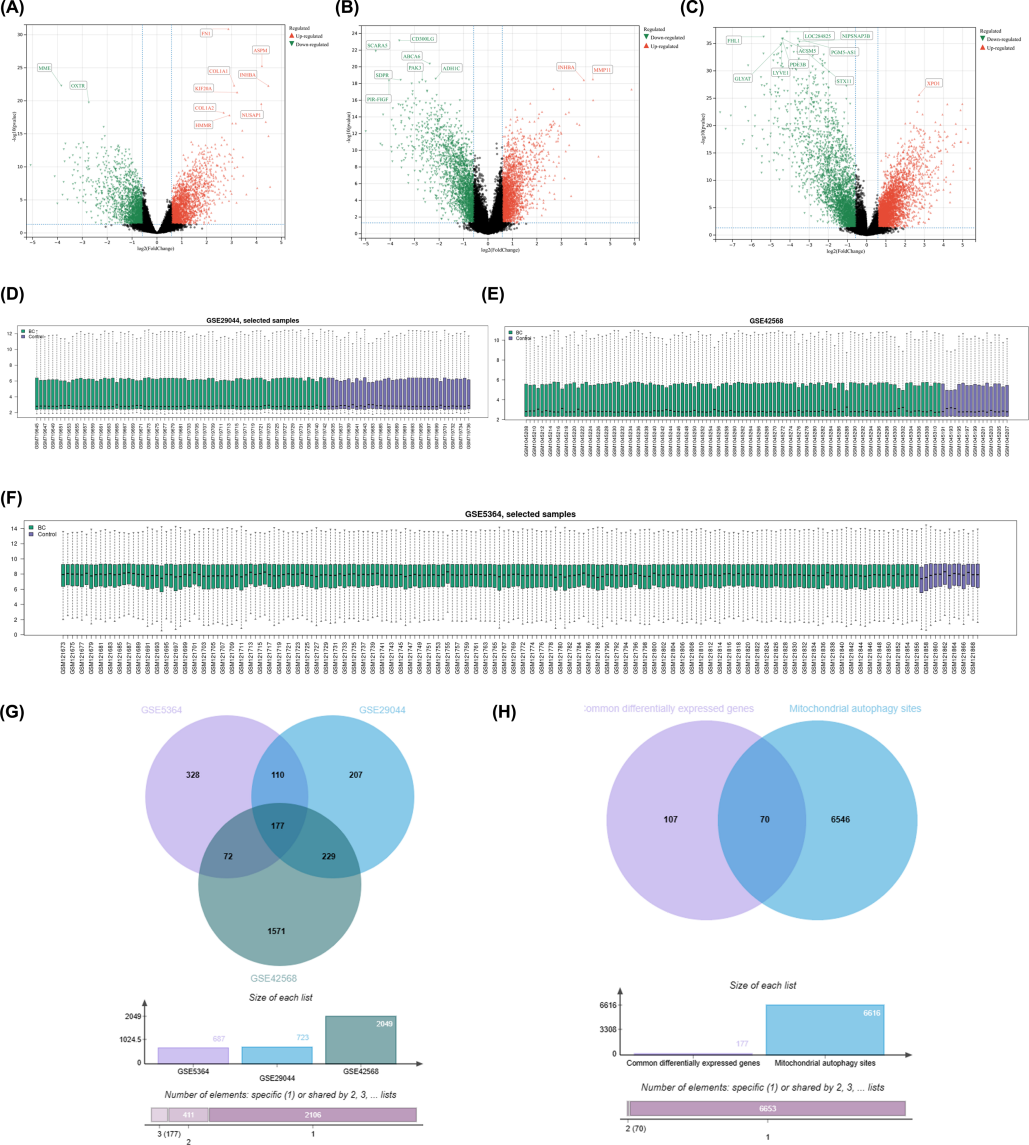
**(A)** Volcano plot of differentially expressed sites in GSE5364; **(B)** Volcano plot of differentially expressed sites in GSE29044; **(C)** Volcano plot of differentially expressed sites in GSE42568; **(D)** Box plot shows well data availability for GSE5364; **(E)** Box plot shows well data availability for GSE29044; **(F)** Box plot shows well data availability for GSE42568; **(G)** Venn diagram shows the intersection of three dataset DEGs; **(H)** Venn diagram shows the intersection between three dataset intersection DEGs and all obtained mitophagy-related sites.

Firstly, three gene expression programs (GSE5364, GSE29044, and GSE42568) from the GEO database were included in our study. We used the website analysis tool GEO2R from GEO database to analyze the transcriptome expression profile data of three datasets to screen DEGs (Log Fold-Change ≥ 1.5 or Log Fold-Change ≤ -1.5 and adj.P<0.05). Among them, in GSE5364, we obtained 687 DEDs, including 401 upregulated sites and 286 downregulated sites; In GSE29044, we obtained 723 DEDs, including 232 upregulated sites and 491 downregulated sites; In GSE42568, we obtained 2049 DEDs, including 966 upregulated sites and 1083 downregulated sites. The DEGs of the three datasets are shown in **Figures 1A–C** by volcanic maps, respectively; The data availability and standardization results are shown in the box plot in **Figures 1D–F**. Subsequently, we intersected three sets of DEGs and obtained 177 common differentially expressed genes (**Figure 1G**). Finally, we downloaded mitophagy-related sites from the GeneCards database, with the inclusion criteria of reliability score>1. And by intersecting 177 common differentially expressed genes with all obtained mitophagy-related sites, 70 mitophagy related sites were ultimately obtained (**Figure 1H**).

### 3.2 Functional cluster analysis for DEGs

**Figure 2:**
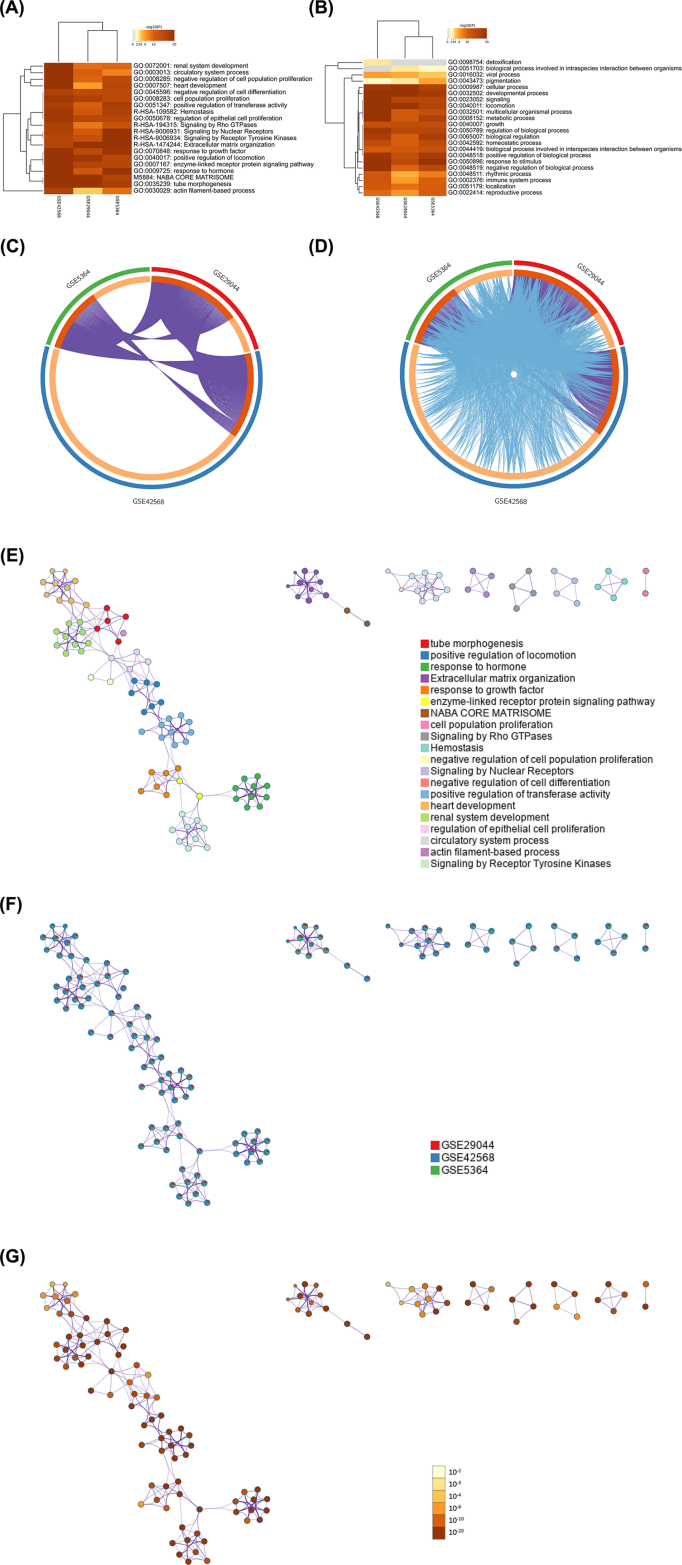
**(A)** Heatmap of enriched terms across input gene lists (colored by p-values) in GO pathway only; **(B)** Heatmap of enriched terms across input gene lists, in all pathway analysis; **(C)** Overlap between gene lists: only at the gene level, where purple curves link identical genes; **(D)** Overlap between gene lists: including the shared term level, where blue curves link genes that belong to the same enriched ontology term; **(E)** Network of enriched terms: Colored by cluster Terms; **(F)** Network of enriched terms: Network of enriched terms represented as pie charts, where pies are color-coded based on the identities of the gene lists; **(G)** Network of enriched terms: Colored by p-value.

In order to understand the potential biological effects and site interactions of DEGs. We used the Metascape database to conduct pathway enrichment and correlation analysis on DEGs. The pathway enrichment analysis in the Metascape database shows that these DEGs are mainly enriched in the following pathways: circulatory system process, negative regulation of cell population proliferation, negative regulation of cell differentiation, cell population proliferation, positive regulation of transferase activity, regulation of epithelial cell proliferation, response to growth factor, positive regulation of locomotion, positive regulation of locomotion, enzyme-linked receptor protein signaling pathway, response to hormone, tube morphogenesis, actin filament-based process from GO terms; and Signaling by Rho GTPases, Signaling by Nuclear Receptors, Signaling by Receptor Tyrosine Kinases, Extracellular matrix organization from Reactome dataset (**Figures 2A, B**). Afterwards, we defined the interaction and similarity relationship of DEGs using the Overlap diagram (**Figures 2C, D**), and the results showed a significant correlation between the significant sites in the three BC datasets. Finally, we constructed a DEGs interaction network through three dimensions (enrichment pathway level, dataset level, and P-value level) to better understand the potential interaction relationships of DEGs (**Figures 2E–G**).

### 3.3 Construction of PPI network for mitophagy-related sites

**Figure 3:**
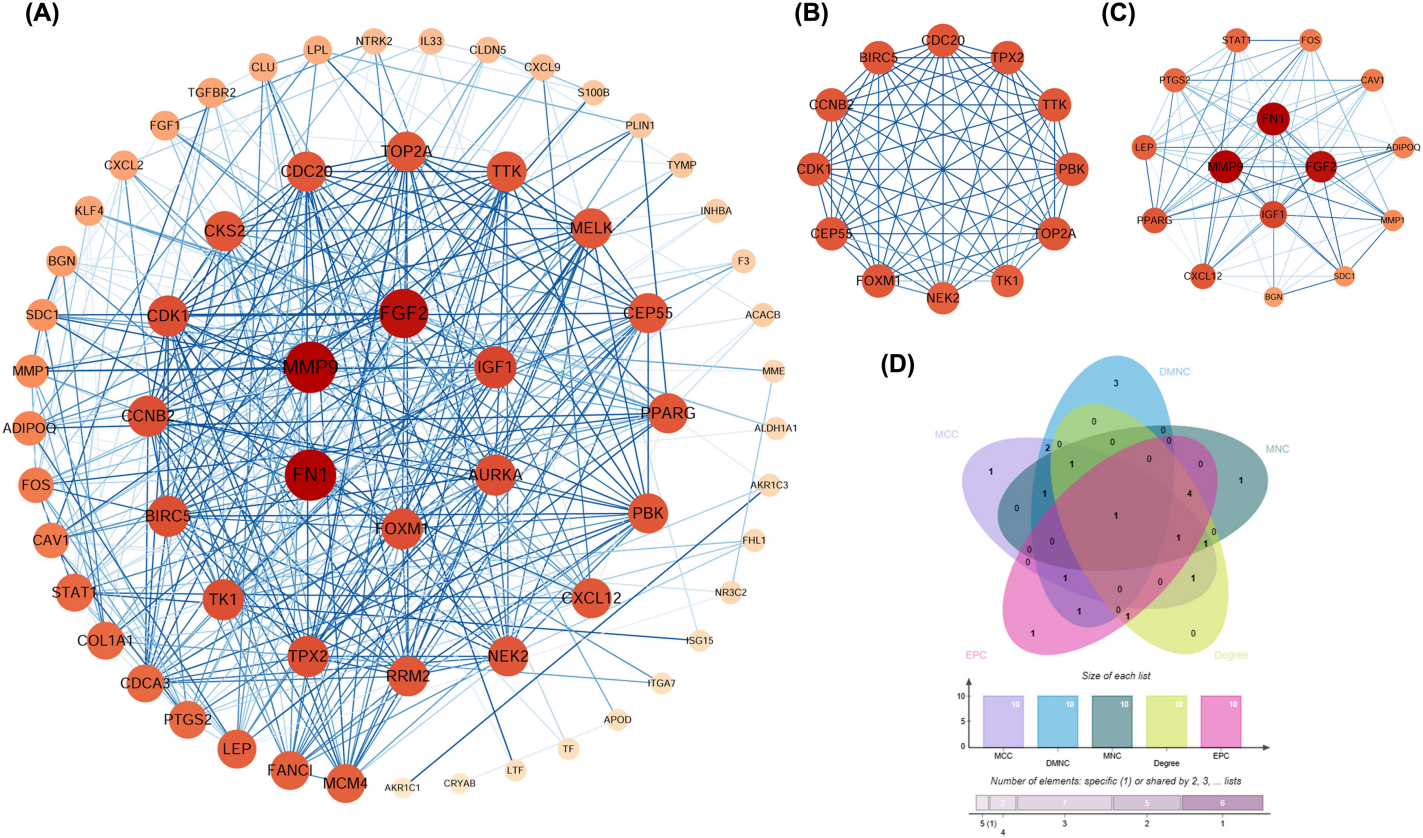
**(A)** PPI networks for 70 mitophagy-related sites with 70 sites and 376 edges; **(B)** Significant network calculated by MCODE plugin: MCODE1; **(C)** Significant network calculated by MCODE plugin: MCODE2; **(D)** Venn plot shows the overlapping hub genes among the five algorithms in the Cytohubba plugin.

To further elucidate the regulation and interaction relationships of 70 mitophagy-related sites, we imported the obtained 70 mitophagy-related sites into the STRING database for protein-protein interaction analysis, and visualized the results in Cytoscape (version 3.8.2). The STRING database identified 70 protein products and 376 edges PPI networks (**Figure 3A**). After that, we used the MCODE plugin in Cytoscape to identify two important interaction modules in the interaction network: MCODE1 and MCODE2 (**Figures 3B, C**). Among them, MCODE1 has obvious interactions, and through the prediction of interaction networks and subnetworks, MCODE1 is believed to play an important role in the development of BC. In addition, we used the cytohubba plugin. The top ten hub genes obtained through five algorithms of MCC, DMNC, MNC, Degree and EPC were shown in **Supplementary Table 1**, and the overlapping hub genes in the five algorithms are verified through the Venn diagram (**Figure 3D**).

### 3.4 Somatic mutation profiles for 70 mitophagy-related sites

**Figure 4:**
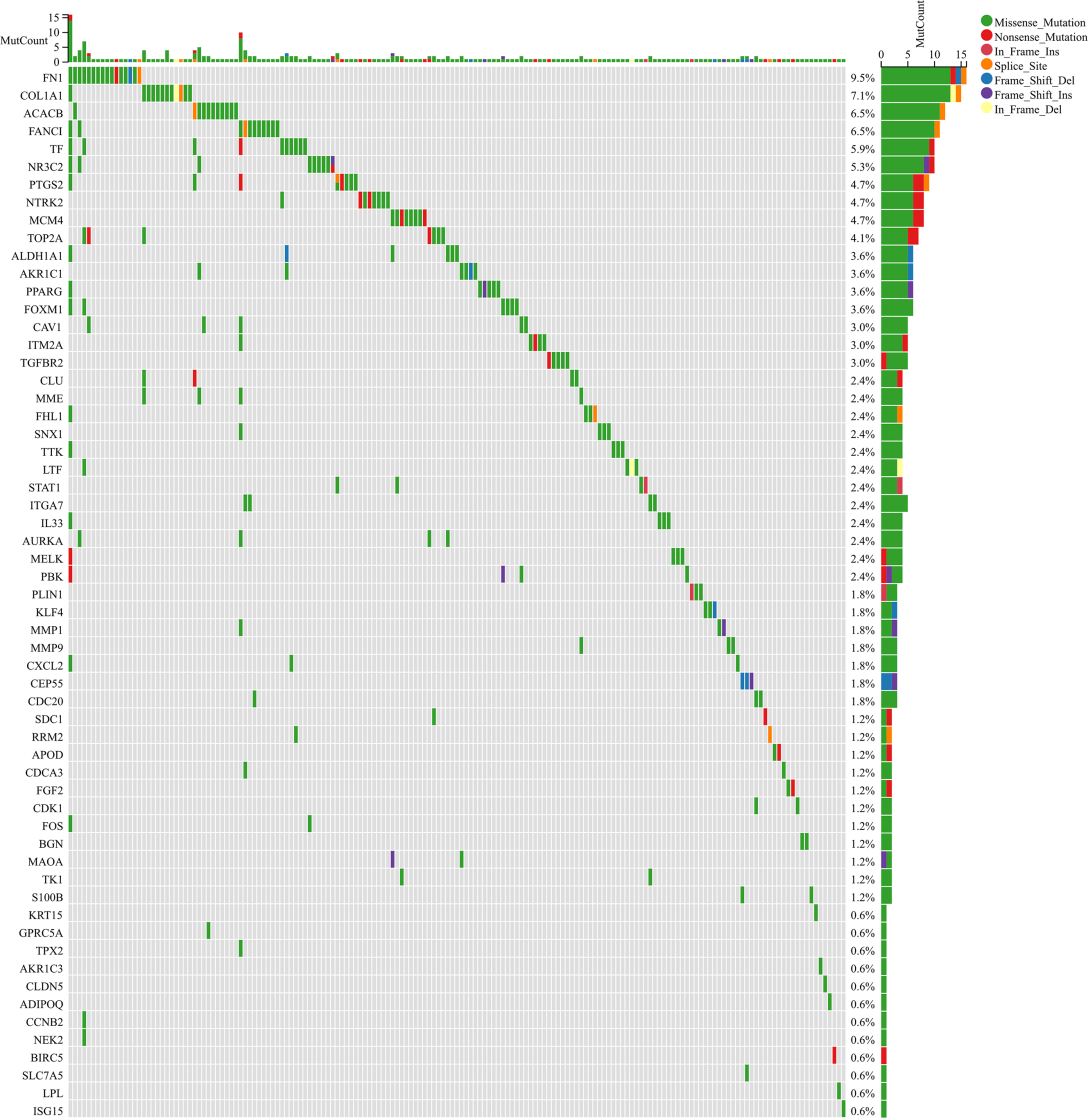
Waterfall diagram shows the comparison of somatic mutation among 70 mitophagy-related sites.

We included 70 mitophagy related sites in the study of somatic mutation. The results showed that in the mutation spectrum data of BC, the mutation degree of 70 mitophagy related sites varied from 9.5% to 0.6% (**Figure 4**), which may be a potential factor leading to the dual role of mitophagy in the prognosis of BC.

### 3.5 Identification of prognostic related sites on survival analysis

**Figure 5:**
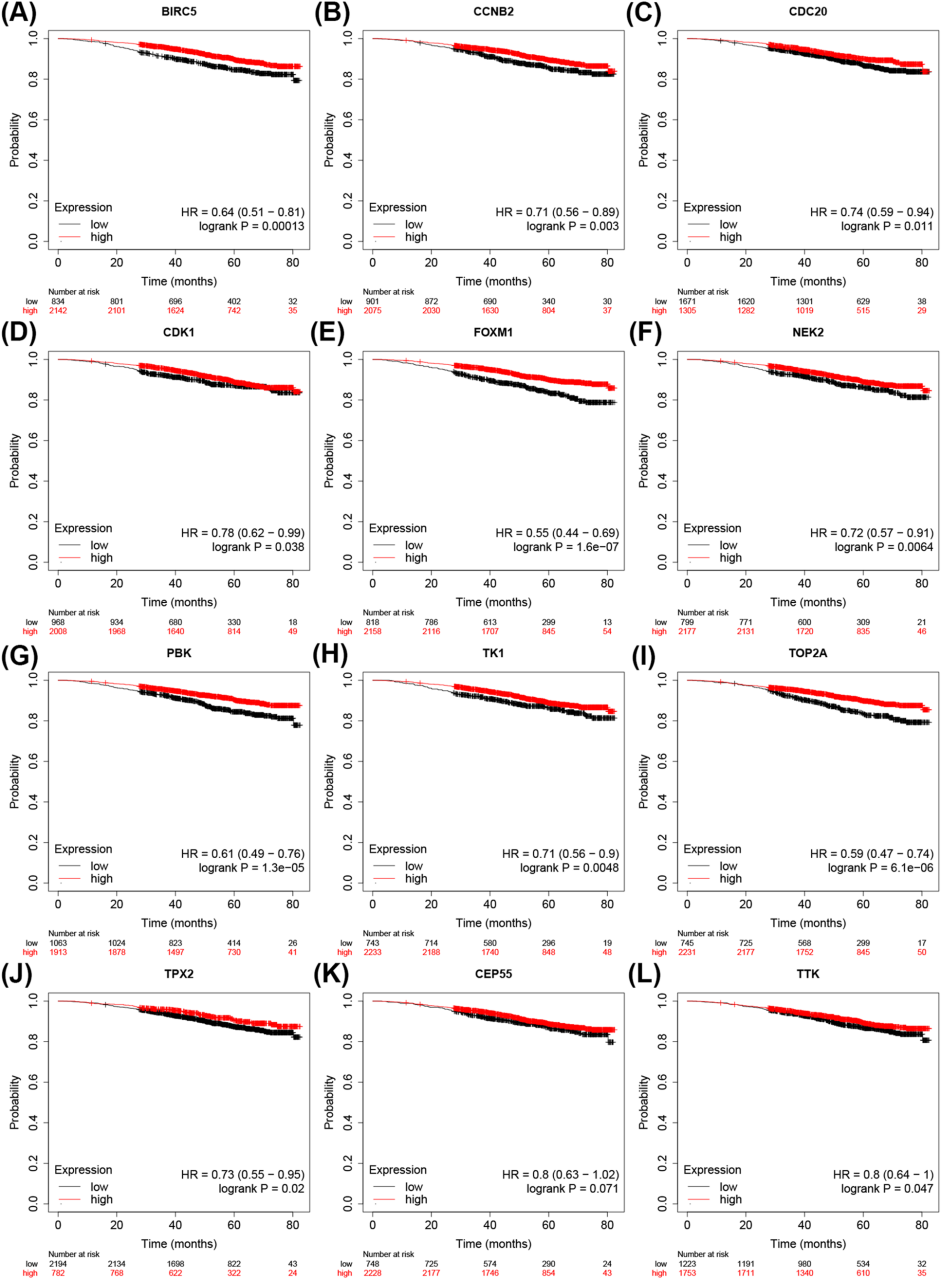
The Kaplan Meier curve reflects the potential prognostic value of mitophagy related sites in BC. **(A–L)** Considered to have value for further prognostic analysis. TTK is also known as CT96; TPX2 is also known as P100; PBK is also known as CT84; CDK1 is also known as CDC2; FOXM1 is also known as TGT3.

The PPI network shows that the interaction between MCODE1 is significant; And through the prediction of MCODE interaction networks and subnetworks, MCODE1 is believed to play an important role in the development of BC. To verify the impact of MCODE1 sites on the prognosis of BC patients and extract potential prognostic biomarkers, we extracted 12 sites from MCODE1 (CDC20, TPX2, TTK, PBK, TOP2A, TK1, NEK2, FOXM1, CEP55, CDK1, CCNB2, BIRC5), and analyzed the prognostic role of each locus in BC based on the Kaplan Meier Plotter database; The inclusion criteria for effective effects are: confidence interval (CI) excluding 1 and logrank-P<0.05. The results showed that 12 sites met the inclusion criteria and were considered valuable for further ROC validation (**Figures 5A–L**).

### 3.6 Confirmation of prognostic sites based on diagnostic and therapeutic efficacy

**Figure 6:**
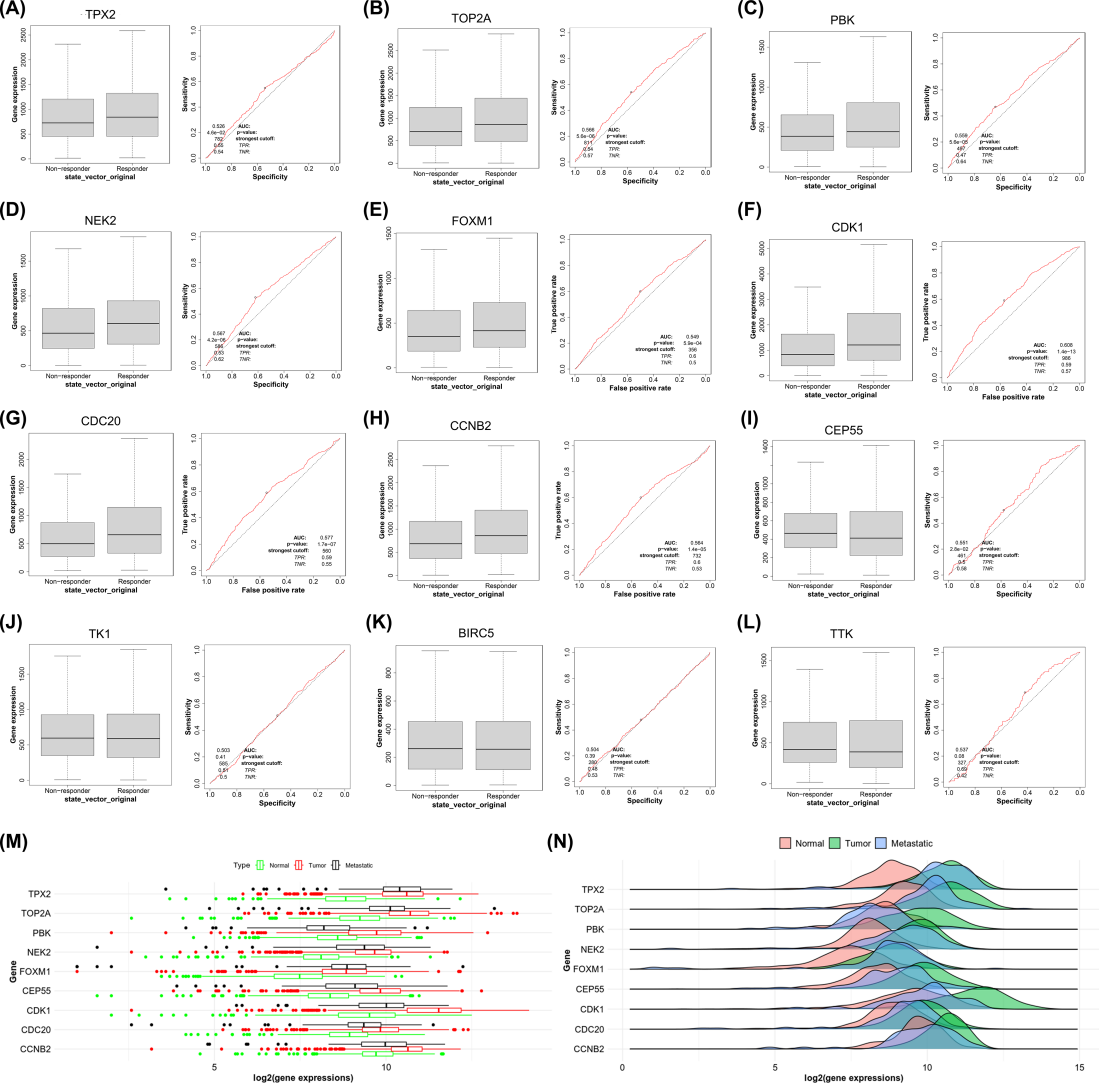
The ROC curve reflects the diagnostic and therapeutic effects of mitophagy-related sites in BC. **(A–I)** Considered to have potential as prognostic biomarkers; **(J–L)** Without prognostic value; **(M, N)** Box plot and ridge plot reflect the expression of 9 prognostic related sites in BC between Control tissues, Tumor tissues, and Metastasis tissues.

For the reason to further confirm the diagnostic and therapeutic efficacy of the 12 sites (CDC20, TPX2, TTK, PBK, TOP2A, TK1, NEK2, FOXM1, CEP55, CDK1, CCNB2, BIRC5) selected in ***“3.5”*** and build a disease diagnosis model based on mitophagy related biomarkers in BC, we use the transcriptome data of BC in the ROC Plotter database, at the same time link gene expression levels with response to therapy, and analyze the prognostic effects of diagnosis and treatment at each site to ultimately determine prognostic biomarkers. The inclusion criteria we adopted were: AUC>0.5, P value<0.05. The final analysis results indicate that 9 sites (CDC20, TPX2, PBK, TOP2A, NEK2, FOXM1, CDK1, CCNB2, CEP55) have potential roles in the diagnosis and treatment of BC, and have the value of becoming biomarkers for BC prognosis (**Figures 6A–L**). Finally, we analyzed the expression of 9 prognostic related sites in control tissue, BC tissue, and BC metastatic tissue, and found that their expression levels in BC tissue were significantly higher than those in control tissue (**Figures 6M, N**).

### 3.7 Consistency cluster analysis identified two mitophagy-BC-related subtypes

**Figure 7:**
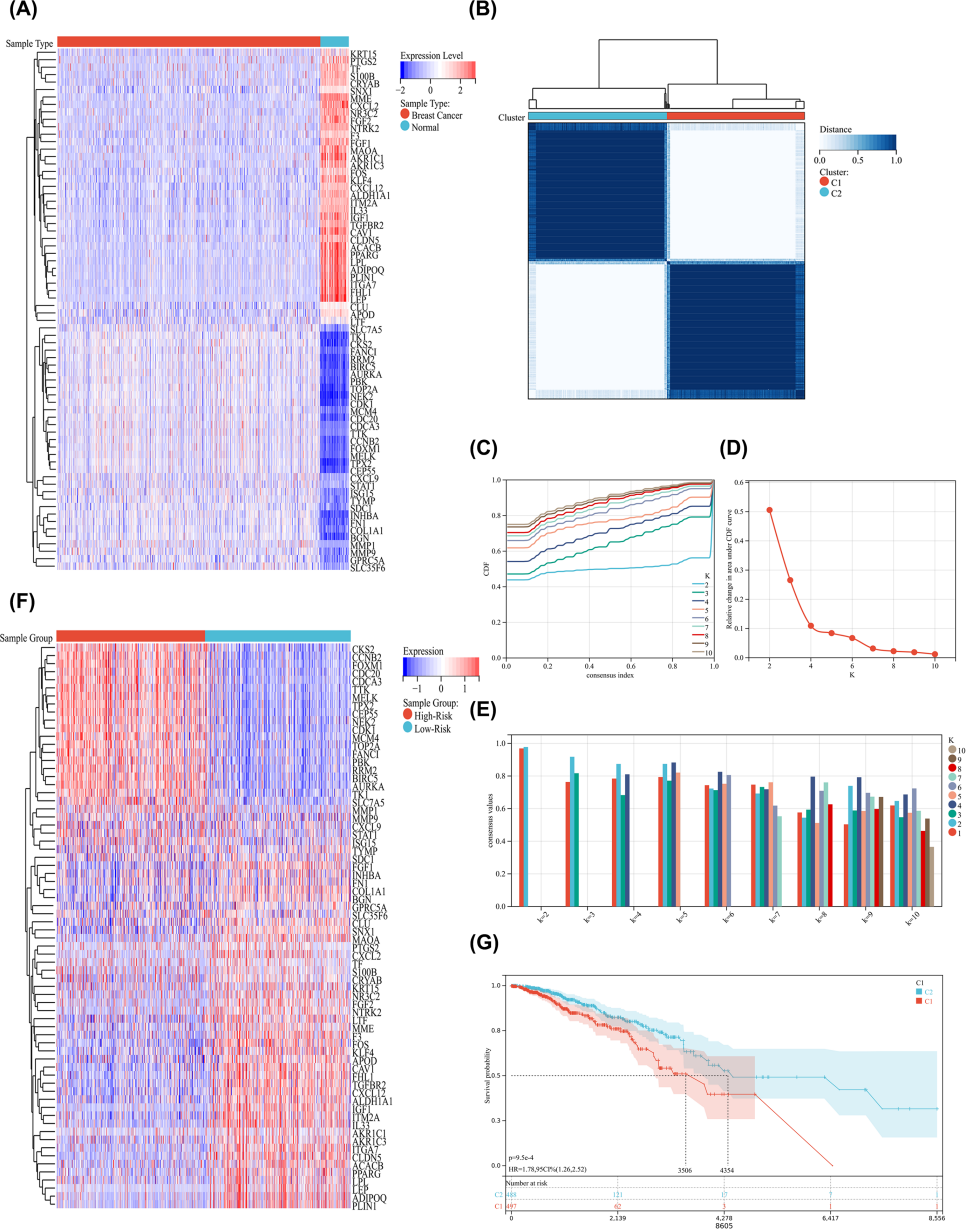
**(A)** Heatmap reflects the differential expression of 70 mitophagy related sites between the BC group and the control group; **(B)** Consistency clustering heatmap reflects subtype grouping; **(C)** Cumulative distribution curve reflects different grouping states; **(D)** The area under the distribution curve reflects the superiority of grouping; **(E)** Sample consistency clustering reflects the scoring values of different clusters; **(F)** Heatmap reflects the differential expression of 70 mitophagy related sites between the High-Risk group and the Low-Risk group; **(G)** K-M survival curve reflects the survival status of the high-risk and low-risk groups.

Firstly, we analyzed the expression differences between BC and control groups for 70 mitophagy related sites based on the transcriptome gene expression profile of TCGA-BRCA project, and the difference results were shown in the form of heatmap (**Figure 7A**). After analyzing the complete body differences, we used Consistency clustering analysis to determine the mitophagy-related clusters of BC samples. After conducting k-means clustering analysis, two subgroups (C1, C2) in the TCGA queue were identified as having different mitophagy gene expression patterns (**Figures 7B–E**). Subsequently, in order to verify the survival status of patients in the C1 and C2 subgroups, we downloaded survival data from the TCGA database for patients in two subgroups and conducted K-M survival analysis on both subgroups. The survival curve showed that the survival level of the C2 subgroup was significantly higher than that of the C1 subgroup (**Figure 7G**). Therefore, we define the C1 subgroup as a high-risk group and the C2 subgroup as a low-risk group. Finally, we analyzed the expression of 70 mitophagy-related sites in the high-risk and low-risk groups, and found significant differences in expression between the two groups (**Figure 7F**). There was a significant block clustering in the heatmap.

### 3.8 GSEA analysis between different subgroups

**Figure 8:**
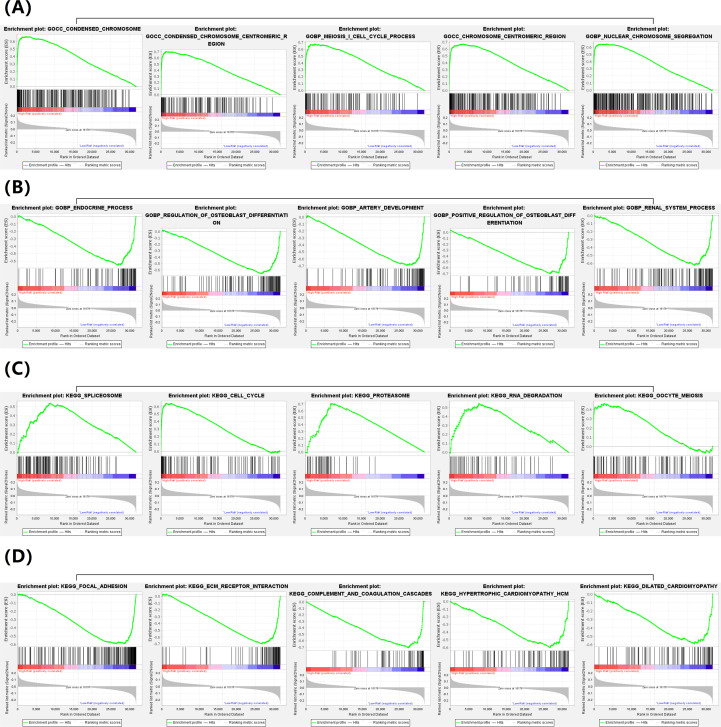
**(A)** Five major enrichment pathways for high-risk groups in GO terms; **(B)** Five major enrichment pathways for low-risk groups in GO terms; **(C)** Five major enrichment pathways for high-risk groups in KEGG terms; **(D)** Five major enrichment pathways for low-risk groups in KEGG terms.

To further determine the potential functional differences caused by differential expression sites in high-risk groups and low-risk groups, we performed a GSEA analysis based on different pathway sets (GO and KEGG) for the transcriptome expression data of the two subgroups. The analysis results show that in GO terms, the condensed chromosome, the condensed chromosome centromeric region, the meiosis i cell cycle process, the chromosome centromeric region, nuclear chromosome segregation are the five major enrichment pathways for high-risk groups (**Figure 8A**); endocrine process, the regulation of osteoblast differentiation, artery development, the positive regulation of osteoblast differentiation, the renal system process are the five major enrichment pathways in the low-risk group (**Figure 8B**). In KEGG terms, spliceosome, cell cycle, proteasome, RNA degradation, oocyte meiosis are the five major enrichment pathways of high-risk groups (**Figure 8C**); focal adhesion, ecm receptor interaction, complement and coagulation cascades, hypertrophic cardiomyopathy hcm, dilated cardiomyopathy are the five major enrichment pathways in the low-risk group (**Figure 8D**).

### 3.9 Immune infiltration analysis and tumor microenvironment recognition

**Figure 9:**
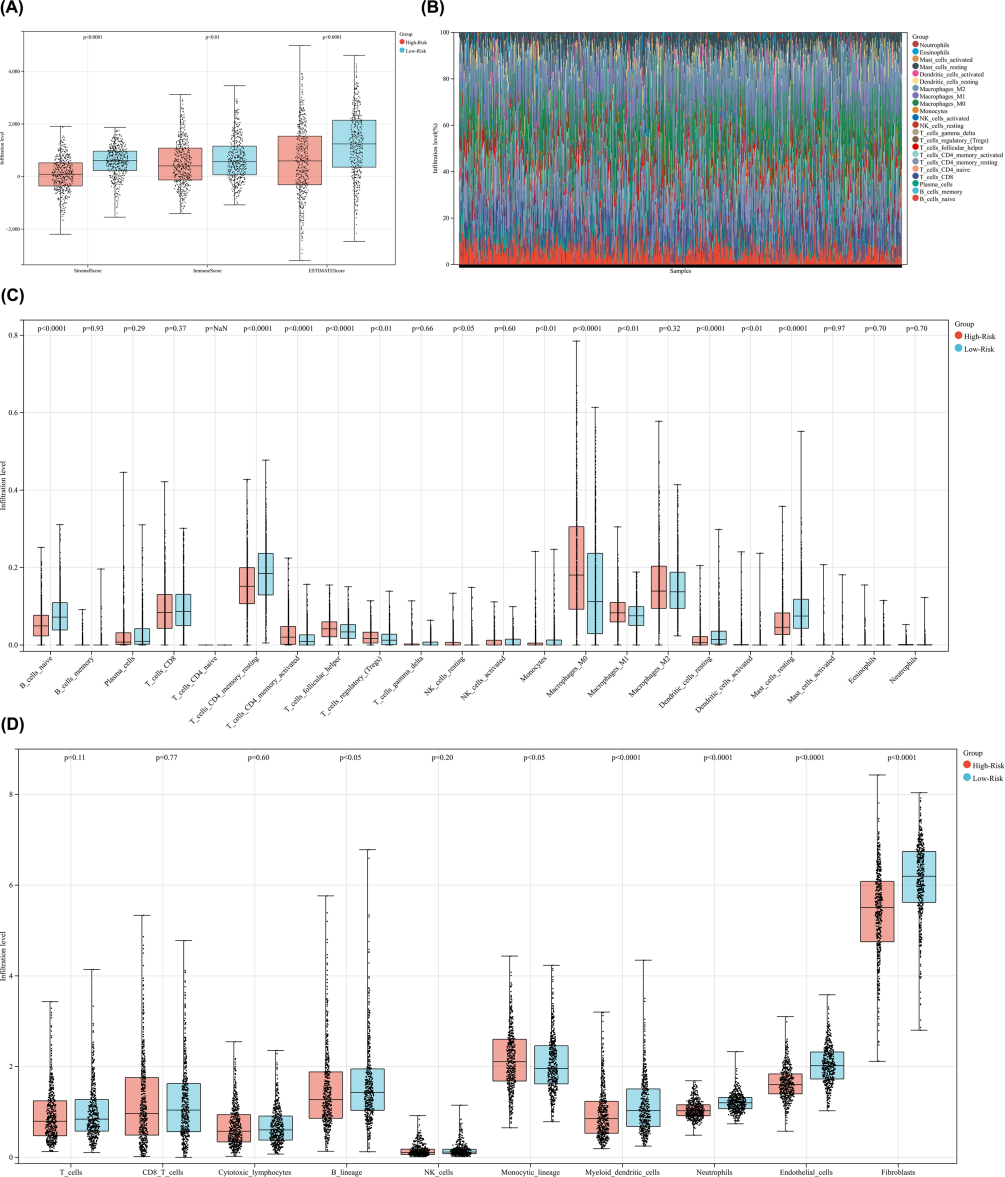
**(A)** Box plot shows the ESTIMATE analysis of immune levels in high-risk and low-risk groups; **(B)** Stacked bar chart showing CIPERSORT immune infiltration analysis; **(C)** Box plot showing CIPERSORT immune infiltration analysis; **(D)** Box plot showing MCPCounter immune infiltration analysis.

We further analyzed the tumor microenvironment and immune invasion level of different subgroups. Firstly, we used the ESTIMATE tool to score the immune levels of the high-risk and low-risk groups. The results showed that the Stromal Score, Immune Score, and ESTIMATE-Score of the low-risk group were higher than those of the high-risk group, and the results were statistically significant (**Figure 9A**). Subsequently, we used the CIBERSORT tool to analyze and validate the infiltration levels of 22 immune cells between the high-risk and low-risk groups. The results showed that, the infiltration level of: cells CD4 memory activated, T cells follicular helper, T cells regulatory (Tregs), NK cells resting, Macrophages M0, Macrophages M1, Dendritic cells increased in the high-risk group; The infiltration level of: B cells native, T cells CD4 memory resting, Monocytes, Dendritic cells resting, Mast cells resting increased in the low-risk group; And the results are all statistically significant. The analysis results are presented in stacked bar charts and box plots (**Figures 9B, C**). Finally, we also used MCPCounter tool for immune infiltration analysis between high-risk and low-risk groups, and the analysis results showed that, the infiltration level of Monocytic lineage increased in the high-risk group; The infiltration level of: B lineage, Myeloid dendritic cells, Neutrophils, Endothelial cells and Fibroblasts increased in the low-risk group; and the results are statistically significant (**Figure 9D**).

### 3.10 Immunohistochemical protein expression validation

**Figure 10:**
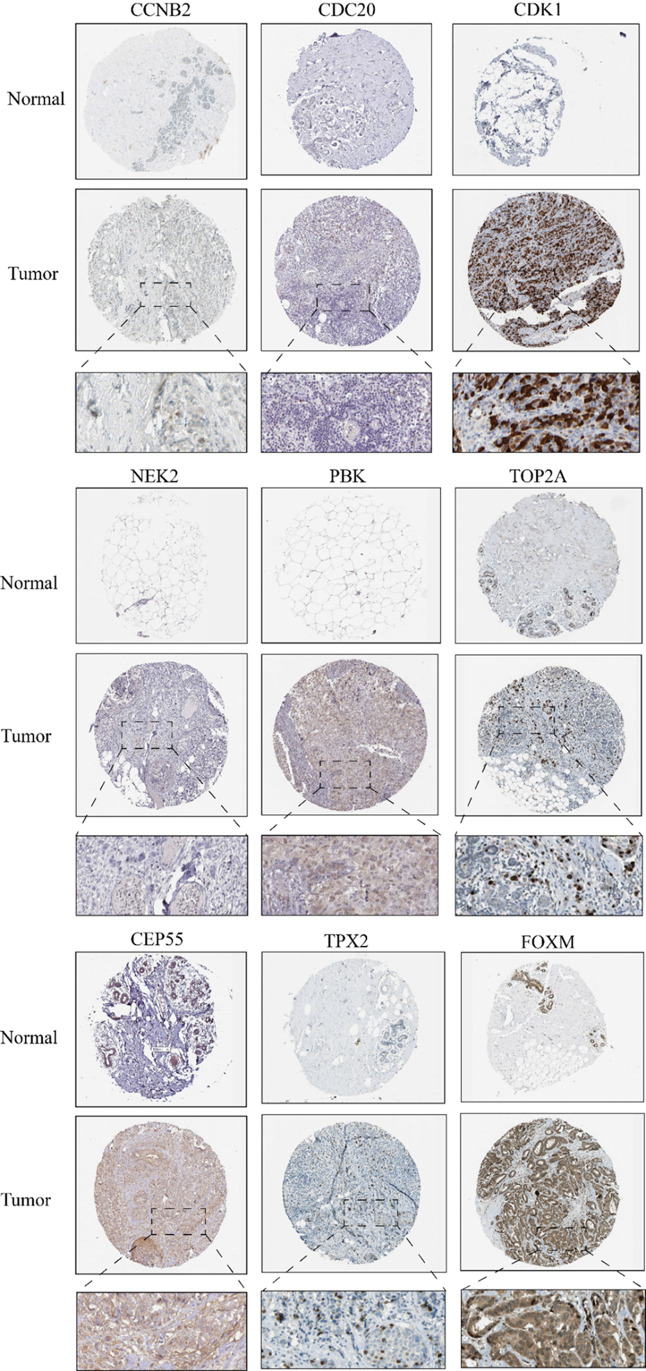
Immunohistochemical images show differences in protein expression of 9 prognostic related sites between BC tissue and control tissue.

We obtained IHC images of the 9 prognostic related sites (CDC20, TPX2, PBK, TOP2A, NEK2, FOXM1, CDK1, CCNB2, CEP55) screened in the Human Protein Atlas database, and further investigated their protein expression differences in normal and BC tissues (**Figure 10**). The results showed that the protein expression of 9 prognostic related sites in BC tissues was higher than that in normal tissues, which was consistent with the above analysis results of transcriptome data.

### 3.11 Functional validation and drug sensitivity predict of mitophagy-related biomarkers in breast cancer

**Figure 11:**
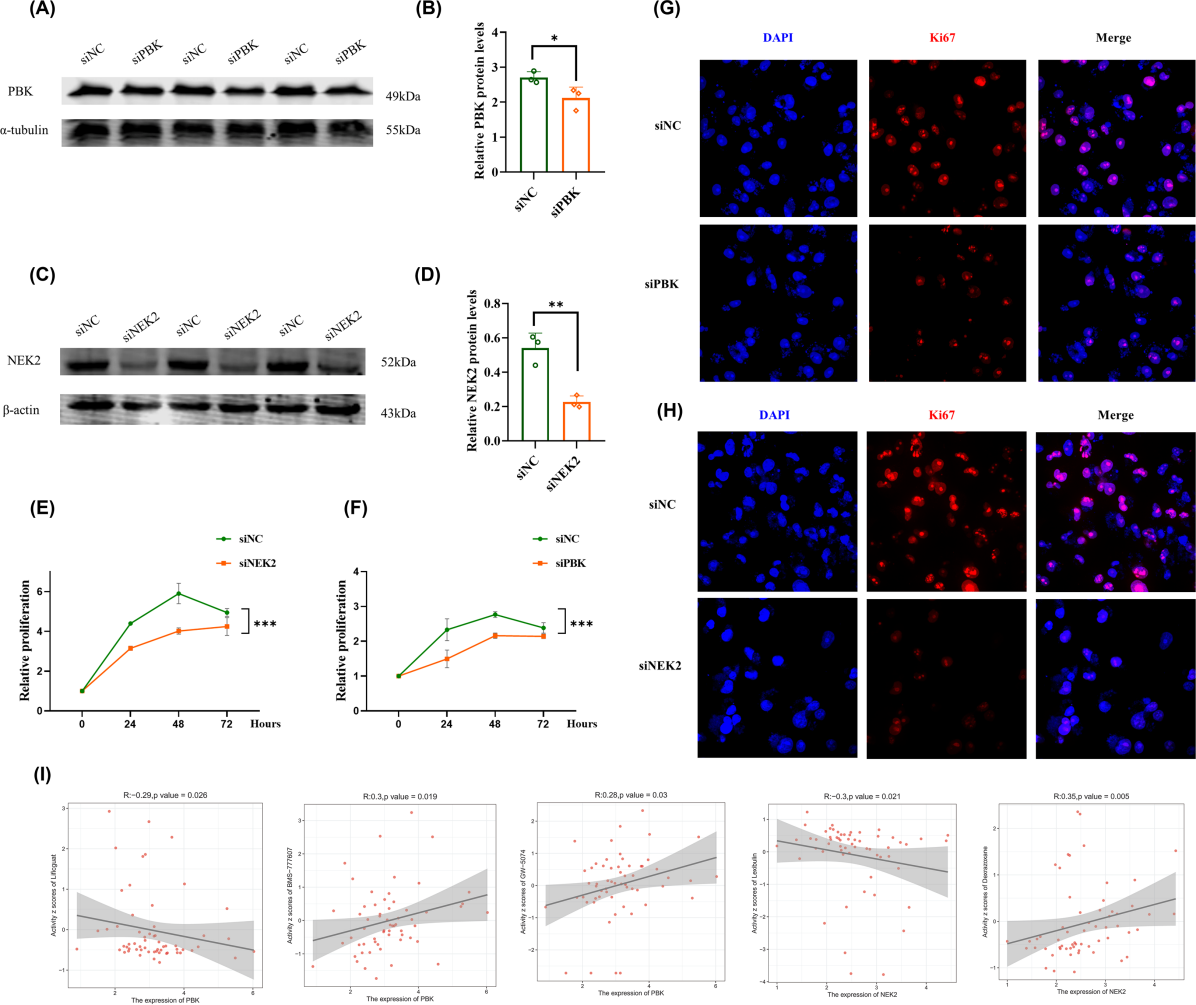
**(A)** Western Blot detects PBK interference in breast cancer cells (Triple-negative breast cancer cells MDA-MB-231); **(B)** Bar chart displaying quantitative expression of PBK protein; **(C)** Western Blot detects NEK2 interference in breast cancer cells (Triple-negative breast cancer cells MDA-MB-231); **(D)** Bar chart displaying quantitative expression of NEK2 protein; **(E)** CCK8 proliferation test to detect the effect of interfering PBK on the proliferation of breast cancer cells (MDA-MB-231); **(F)** CCK8 proliferation test to detect the effect of interference with NEK2 on the proliferation of breast cancer cells (MDA-MB-231); **(G)** Confocal to detect the effect of PBK interference on the proliferation of breast cancer cells (MDA-MB-231); **(H)** Confocal to detect the effect of interference with NEK2 on the proliferation of breast cancer cells (MDA-MB-231). **(I)** Drug prediction analysis targeting specific targets.

**Figure 12:**
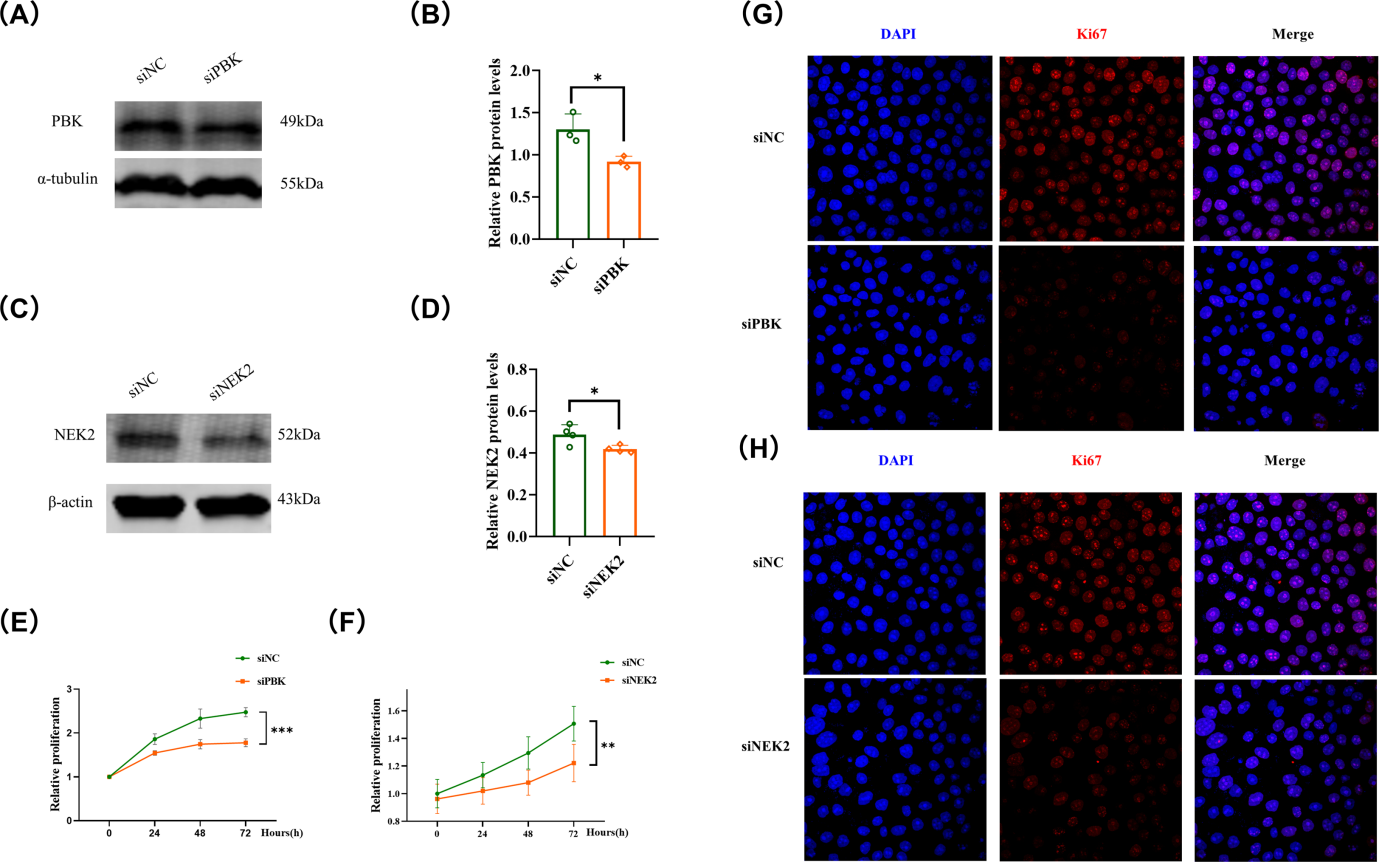
**(A)** Western Blot detects PBK interference in breast cancer cells (Non triple negative breast cancer cells MCF-7); **(B)** Bar chart displaying quantitative expression of PBK protein; **(C)** Western Blot detects NEK2 interference in breast cancer cells (Non triple negative breast cancer cells MCF-7); **(D)** Bar chart displaying quantitative expression of NEK2 protein; **(E)** CCK8 proliferation test to detect the effect of interfering PBK on the proliferation of breast cancer cells (MCF-7); **(F)** CCK8 proliferation test to detect the effect of interference with NEK2 on the proliferation of breast cancer cells (MCF-7); **(G)** Confocal to detect the effect of PBK interference on the proliferation of breast cancer cells (MCF-7); **(H)** Confocal to detect the effect of interference with NEK2 on the proliferation of breast cancer cells (MCF-7).

To further validate the biological functions of the mitophagy-related prognostic biomarkers identified above, we selected PBK and NEK2, two genes that exhibited the most significant prognostic value and expression differences, for functional experiments in breast cancer cells (Triple-negative breast cancer cells MDA-MB-231). Western blot analysis confirmed that siRNA-mediated knockdown of PBK and NEK2 markedly reduced their protein expression levels compared with the control group (**Figures 11A–D**). Subsequently, CCK-8 assays demonstrated that silencing either PBK or NEK2 significantly inhibited the proliferation of breast cancer cells (**Figures 11E, F**). Moreover, confocal microscopy analysis revealed that the proliferation activity and nuclear density of breast cancer cells were notably decreased after PBK or NEK2 interference (**Figures 11G, H**). PBK is negatively correlated with the compound Lificguat and positively correlated with the compound GW-5074 and BMS-777607 (P<0.05), while NEK2 is negatively correlated with the compound Lexibulin and positively correlated with the drug Dexrazoxane (P<0.05) (**Figure 11I**). Furthermore, in order to further verify the stability of the results, we further verified the functions of PBK and NEK2 in the Non triple negative breast cancer cell line MCF-7, and got the same results (**Figures 12A–H**).

### 3.12 Clinicopathological characteristics and risk-associated factors

Clinicopathological characteristics of patients in the high- and low-risk groups are summarized in **Supplementary Table 2**, with categorical variables compared using the χ² test. Survival status did not differ significantly between groups (P = 0.512). Significant differences were observed in T stage (P < 0.001), M stage (P = 0.002), and clinical stage (P < 0.001). The high-risk group showed a higher proportion of T2 tumors, more M0 cases, and increased proportions of stage IIA and IIB, while fewer early-stage tumors were observed, indicating more advanced tumor characteristics. N stage showed a marginal difference (P = 0.051). Continuous variables were analyzed using independent-samples t tests (**Supplementary Table 3**). Patients in the high-risk group were slightly younger than those in the low-risk group (56.61 vs. 58.74 years; mean difference = −2.13; P = 0.009). In addition, PBK and NEK2 expression levels were significantly higher in the high-risk group (both P < 0.001). Logistic regression analyses were performed to assess risk-associated factors (**Supplementary Table 4**). In univariate analysis, PBK and NEK2 were strongly associated with high-risk status (both P < 0.001). After mutual adjustment, both PBK (OR = 3.23, 95% CI: 2.43–4.28) and NEK2 (OR = 3.37, 95% CI: 2.52–4.51) remained independently associated with high-risk status, supporting their roles as robust contributors to risk stratification.

## 4 Discussion

Mitophagy regulatory factors have extensive interactions and are involved in the occurrence and development of various diseases, and are closely related to various cellular behaviors; Among them, multiple studies have shown that mitophagy may play an important role in inflammatory response, immune regulation, and tumor progression (28, 29). Although there have been many studies reflecting the important role of mitophagy in tumors, most studies are based on individual sites related to mitophagy, exploring their regulatory role in tumor occurrence and development. The characteristics of the overall regulatory role of multiple mitophagy related sites in tumors have not been fully elucidated (30–32). Therefore, identifying prognostic biomarkers related to mitophagy and clarifying the potential role of mitophagy in BC will deepen our understanding of the occurrence and development of BC, and provide new insights for the innovation of early diagnosis and treatment strategies for BC.

In our research, we first identified mitophagy-related sites using multiple transcriptome datasets, and then constructed a PPI network for the obtained sites to ensure the interaction between the localization points, so as to perform the preliminary screening of prognostic related sites; Subsequently, we utilized survival analysis and diagnostic and therapeutic efficacy prediction to further screen 12 selected mitophagy-related sites, and identified 8 prognostic biomarkers for mitophagy-related BC. Gene expression profiles and IHC confirmed that the mRNA and protein expression levels of 9 sites (CDC20, TPX2, PBK, TOP2A, NEK2, FOXM1, CDK1, CCNB2, CEP55) in BC were higher than those in the control group. In addition, we identified two prognostic subtypes in BC based on mitophagy related sites. Through GSEA analysis and immune infiltration analysis, we further identified the characteristics of the two prognostic subtypes; A noteworthy result is that the degree of immune infiltration in the low-risk subtype is higher than that in the high-risk subtype, which may be a potential influencing factor for a better prognosis in the low-risk subtype.

Mitophagy is crucial for regulating tumor cells and is directly or indirectly related to the development of tumors. Firstly, mitophagy is closely related to maintaining mitochondrial and cellular homeostasis (33). Due to the accumulation of damaged and defective mitochondria involved in carcinogenesis, it plays an important role in a tumor inhibition mechanism (34); Lauren et al. found that mitophagy inhibits tumor growth by removing dysfunctional mitochondria; otherwise, abnormal mitochondria may alter cells and promote tumor development (35). Other research results indicate that in malignant tumors, mitophagy also involves abnormal activation and proliferation of cancer cells (36). This indicates that it can exert both carcinogenic and tumor suppressive effects simultaneously. The balance between the two effects can determine tumor progression or apoptosis, and the differential expression of different mitophagy regulatory factors may have a potential impact on tumor prognosis (37). Recent studies have further expanded the understanding of the dual role of mitophagy in cancer, suggesting that its biological effects are highly context-dependent and dynamically regulated (38). While mitophagy can suppress tumor initiation by preserving mitochondrial integrity and limiting oxidative stress, accumulating evidence indicates that, in established tumors, enhanced or dysregulated mitophagy may promote cancer cell survival, metabolic flexibility, and proliferation under adverse conditions (39). Moreover, mitophagy has been shown to participate in therapy adaptation and modulation of the tumor immune microenvironment, thereby contributing to tumor progression and treatment resistance. Importantly, these opposing effects appear to be largely determined by the differential expression and functional dominance of specific mitophagy regulatory factors, underscoring the complexity of mitophagy regulation in cancer and its potential impact on clinical outcomes (40, 41). In this study, we preliminarily identified 9 prognostic biomarkers related to mitophagy in BC (CDC20, TPX2, PBK, TOP2A, NEK2, FOXM1, CDK1, CCNB2, CEP55).

Existing research suggests that these 9 sites play a crucial role in BC. Cell division cycle 20 (CDC20) is a key regulatory factor for cell division. Abnormal expression of CDC20 is associated with premature development promotion, leading to abnormal mitosis and playing an important role in formation and metastasis (42, 43). The target protein for Xenopus kinesin-like protein 2 (TPX2) is a prohibitin strictly regulated by cell cycle, with involvement in microtubule-associated proteins formed by spindle apparatus in mitosis;Significantly up-regulated TPX2 expression was observed in breast cancer tissues and cells, and proliferation, migration and invasion of breast cancer cells (44, 45). PDZ binding kinase (PBK) is a new serine threonine kinase, a member of the mitogen activated protein kinase (MAPK) family, which can be used as an upstream kinase to transform oncogenes in some key signal pathways; Research shows that the prognosis of breast cancer patients with high expression of PBK is poor (46, 47). Topoisomerase2α (TOP2A) is a ribozyme encoded by chromosome 17 that regulates topological changes in DNA by promoting transient double stranded DNA cleavage; high expression of TOP2A helps identify BC patients with high risk of recurrence (48, 49). NIMA Related Kinase 2 (NEK2) is a mitotic kinase that is frequently upregulated in human cancer, contributing to the proliferation of malignant tumors and inducing drug resistance; Research has shown that NEK2 can promote the proliferation and invasion of BC (50, 51). Transcription factor forkhead box M1 (FoxM1) is overexpressed in breast cancer, which is associated with poor prognosis; In addition, FoxM1 can also collaborate with many cancer promoting signaling pathways to drive the invasive progression of tumors (52, 53). Cyclin dependent kinase 1 (CDK1) is a serine/threonine kinase that can co-regulate cell cycle with certain cyclin regulating proteins; A large amount of evidence suggests that high expression of CDK1 is significantly associated with poor prognosis in various tumors (54, 55). Cyclin B2 (CCNB2) is a member of the Cyclin B family, which plays a role in G2/M transition through cell division control (CDC2) activation and exerts inhibitory effects, leading to cell cycle arrest. The research shows that the high expression of CCNB2 can promote the migration of BC cell migration and enhance the invasion of BC (56, 57). Centrosome protein 55 (CEP55) is a key regulator of cell division. Its overexpression is related to genomic instability, which is a sign of cancer; Moreover, CEP55 is considered an important risk factor for adverse clinical outcomes in BC (58). In summary, various research data indicate that 9 diagnostic biomarkers related to mitophagy represent a poor prognosis for BC and have great value as early diagnostic biomarkers for BC.

Emerging evidence indicates that mitophagy in tumor cells actively modulates the tumor immune microenvironment, including in BC. Excessive mitophagy in breast cancer cells has been shown to suppress chemokine secretion, such as CCL2, leading to reduced recruitment of immunosuppressive cells, including tumor-associated macrophages (TAMs), regulatory T cells (Tregs), and myeloid-derived suppressor cells (MDSCs), and consequently enhancing CD8^+^ T-cell cytotoxic activity (59). At the molecular level, mitophagy can influence immune checkpoint regulation, such as PD-L1 expression, thereby further shaping immune cell infiltration and anti-tumor immune responses. Mechanistically, these effects are mediated through mitophagy-dependent modulation of mitochondrial stress signaling pathways, including the cGAS–STING axis, NF-κB–driven inflammatory signaling, and hypoxia-associated HIF-1α pathways, which collectively regulate chemokine production and immune cell recruitment (40, 60). Besides, bioinformatic analyses have revealed distinct immune infiltration patterns associated with different mitophagy-related gene expression profiles in BC. Together, these findings suggest that tumor cell mitophagy can regulate immune cell recruitment and activation through coordinated effects on chemokine signaling, immune checkpoints, and immune-related pathways within the tumor microenvironment (38, 61). In addition, our study also identified a noteworthy aspect. The study of immune infiltration in the BC subgroups shows that: firstly, the three ESTIMATE scores of the low-risk subgroup are higher, indicating a stronger immune microenvironment in the low-risk group. Secondly, MCPCounter immune infiltration analysis revealed that in the low-risk subgroup, the infiltration levels of immune and stromal cells such as B lineage, myeloid dendritic cells, neutrophils, endothelial cells, and fibroblasts were higher (62–65). These results suggest that higher immune cell infiltration may contribute to a better prognosis in the low-risk subgroup Based on this, we propose a hypothesis: in breast cancer, mitophagy may affect the prognosis of breast cancer patients by changing the infiltration of immune cells.

Moreover, to experimentally validate the functional relevance of the mitophagy-related biomarkers, we performed *in vitro* assays focusing on PBK and NEK2, two hub genes that showed strong prognostic value and high expression in breast cancer. Knockdown of PBK or NEK2 significantly suppressed the proliferation of breast cancer cells, as confirmed by Western blot, CCK-8, and confocal microscopy assays. These results indicate that PBK and NEK2 may act as oncogenic drivers in breast cancer by promoting cell proliferation and survival, supporting their roles as critical components in the mitophagy-related regulatory network. Considering their dual roles in both mitophagy regulation and tumor progression, PBK and NEK2 may represent promising therapeutic targets for breast cancer treatment. In addition, our drug sensitivity analysis results also revealed that for PBK, its expression level is negatively correlated with Lificguat molecules; For NEK2, its expression level is negatively correlated with Lexibulin molecules, reflecting the potential of Lificguat and Lexibulin as potential molecules for BC treatment.

Collectively, our study provides a new perspective for understanding the molecular mechanisms underlying breast cancer progression. Through multidimensional bioinformatics and experimental validation, we identified nine mitophagy-related prognostic biomarkers, among which PBK and NEK2 were further confirmed to enhance breast cancer cell proliferation. Additionally, by stratifying patients into high- and low-risk subgroups, we demonstrated that mitophagy-related gene expression patterns are associated with distinct immune infiltration and pathway enrichment profiles. Taken together, our findings suggest that mitophagy may influence breast cancer prognosis by modulating immune cell infiltration and tumor cell proliferative capacity. Although this study lacks large-scale molecular and clinical validation, it provides a robust foundation for further mechanistic exploration and translational research. In the future, integrating mitophagy-based biomarkers such as PBK and NEK2 into clinical evaluation may contribute to early diagnosis, individualized therapy, and improved prognosis in breast cancer patients.

## Data Availability

The raw data supporting the conclusions of this article will be made available by the authors, without undue reservation.
