# Supplementary material for: Comprehensive analysis of mitophagy-related genes reveals prognostic signatures in breast cancer: based on immune landscapes and treatment target predict
Source: Front Immunol. 2026 Feb 5;17:1740830. doi: 10.3389/fimmu.2026.1740830 (PMC12916666; doi:10.3389/fimmu.2026.1740830)
Supplement: Supplementary file 1 [file Table1.docx]

**sTable 1.** Hub genes among the five algorithms in the Cytohubba plugin.

| MCC | DMNC | MNC | Degree | EPC |
| --- | --- | --- | --- | --- |
| TPX2 | CDC20 | FN1 | MMP9 | MMP9 |
| BIRC5 | CCNB2 | MMP9 | FN1 | FGF2 |
| CDK1 | TOP2A | FGF2 | FGF2 | FN1 |
| CCNB2 | TTK | IGF1 | IGF1 | FOXM1 |
| FOXM1 | MELK | FOXM1 | TPX2 | CXCL12 |
| AURKA | CEP55 | BIRC5 | BIRC5 | IGF1 |
| CDC20 | PBK | CDK1 | CCNB2 | PBK |
| TOP2A | FOXM1 | PPARG | CDK1 | MELK |
| TTK | RRM2 | CDC20 | FOXM1 | TK1 |
| MELK | CKS2 | CCNB2 | TK1 | BIRC5 |

**sTable 2.** Clinicopathological characteristics of patients stratified by the PBK–NEK2 risk model.

| **Term** | **High-Risk** | | **Low-Risk** | | **χ2** | **P-Value** |
| --- | --- | --- | --- | --- | --- | --- |
|  | **Counts** | **% (High-risk group)** | **Counts** | **% (Low-risk group)** |  |  |
| Survival-Status |  | | | | 0.455 | 0.512 |
| Survive | 406 | 85.7 | 427 | 87.1 |  | |
| Death | 68 | 14.3 | 63 | 12.9 |  |  |
| T-Stage |  | | | | 40.42 | **<0.001** |
| T1 | 15 | 3.2 | 25 | 5.1 |  | |
| T1a | 0 | 0 | 1 | 0.2 |  |  |
| T1b | 3 | 0.6 | 12 | 2.4 |  |  |
| T1c | 74 | 15.6 | 128 | 26.1 |  |  |
| T2 | 306 | 64.6 | 246 | 50.2 |  |  |
| T2a | 0 | 0 | 1 | 0.2 |  |  |
| T2b | 1 | 0.2 | 0 | 0 |  |  |
| T3 | 54 | 11.4 | 65 | 13.3 |  |  |
| T3a | 0 | 0 | 1 | 0.2 |  |  |
| T3b | 1 | 0.2 | 0 | 0 |  |  |
| T4 | 3 | 0.6 | 3 | 0.6 |  |  |
| T4b | 12 | 2.5 | 8 | 1.6 |  |  |
| T4d | 2 | 0.4 | 0 | 0 |  |  |
| TX | 3 | 0.6 | 0 | 0 |  |  |
| N-Stage |  | | | | 23.568 | 0.051 |
| N0 | 142 | 30 | 139 | 28.4 |  | |
| N0 (i-) | 72 | 15.2 | 68 | 13.9 |  |  |
| N0 (i+) | 6 | 1.3 | 20 | 4.1 |  |  |
| N0 (mol+) | 0 | 0 | 1 | 0.2 |  |  |
| N1 | 62 | 13.1 | 46 | 9.4 |  |  |
| N1a | 75 | 15.8 | 81 | 16.5 |  |  |
| N1b | 14 | 3 | 17 | 3.5 |  |  |
| N1mi | 12 | 2.5 | 19 | 3.9 |  |  |
| N2 | 30 | 6.3 | 20 | 4.1 |  |  |
| N2a | 32 | 6.8 | 28 | 5.7 |  |  |
| N3 | 9 | 1.9 | 16 | 3.3 |  |  |
| N3a | 12 | 2.5 | 26 | 5.3 |  |  |
| N3b | 1 | 0.2 | 1 | 0.2 |  |  |
| N3c | 1 | 0.2 | 0 | 0 |  |  |
| NX | 6 | 1.3 | 8 | 1.6 |  |  |
| M-Stage |  | | | | 12.359 | **0.002** |
| M0 | 407 | 85.9 | 398 | 81.2 |  | |
| M1 | 14 | 3 | 5 | 1 |  |  |
| MX | 53 | 11.2 | 87 | 17.8 |  |  |
| Clinical-Stage |  | | | | 34.996 | **<0.001** |
| Stage I | 25 | 5.3 | 56 | 11.1 |  | |
| Stage IA | 28 | 5.9 | 54 | 11 |  |  |
| Stage IB | 3 | 0.6 | 3 | 0.6 |  |  |
| Stage II | 2 | 0.4 | 3 | 0.6 |  |  |
| Stage IIA | 170 | 35.9 | 139 | 28.4 |  |  |
| Stage IIB | 122 | 25.7 | 109 | 22.2 |  |  |
| Stage III | 1 | 0.2 | 1 | 0.2 |  |  |
| Stage IIIA | 73 | 15.4 | 69 | 14.1 |  |  |
| Stage IIIB | 12 | 2.5 | 7 | 1.4 |  |  |
| Stage IIIC | 20 | 4.2 | 39 | 8 |  |  |
| Stage IIIC | 13 | 2.7 | 5 | 1 |  |  |
| Stage IV | 5 | 1.1 | 5 | 1.1 |  |  |

**sTable 3.** Comparison of continuous clinical variables and model-related gene expression between risk groups.

| **Term** | **High-Risk** | | **Low-Risk** | | **Mean difference** | **95%CI** | **t** | **P-Value** |
| --- | --- | --- | --- | --- | --- | --- | --- | --- |
|  | **Counts** | **Average Value** | **Counts** | **Average Value** |  |  |  |  |
| Age | 474 | 56.61 | 490 | 58.74 | -2.127 | -3.731 - -0.523 | -2.602 | **0.009** |
| PBK | 474 | 3.33 | 490 | 2.08 | 1.25 | 1.148 - 1.351 | 24.052 | **<0.001** |
| NEK2 | 474 | 3.64 | 490 | 2.41 | 1.23 | 1.129 - 1.33 | 24.001 | **<0.001** |

**sTable 4.** Univariate and multivariate logistic regression analyses identifying predictors of high-risk classification.

| **Term** | **B** | **SE** | **Wald** | **OR** | **95% CI for OR** | **P value** |
| --- | --- | --- | --- | --- | --- | --- |
| Univariate Model |  | | | | | |
| PBK | 1.902 | 0.123 | 239.74 | 6.696 | 5.264-8.519 | <0.001 |
| NEK2 | 1.951 | 0.127 | 235.44 | 7.036 | 5.484-9.027 | <0.001 |
| Multivariate Model ^†^ |  | | | | | |
| PBK | 1.172 | 0.144 | 66.183 | 3.228 | 2.434-4.281 | <0.001 |
| NEK2 | 1.216 | 0.149 | 66.829 | 3.372 | 2.520-4.514 | <0.001 |

*† Multivariate model included PBK and NEK2 simultaneously.*
